# A Cross-National Comparison on Subjective Well-Being of Kindergarten Teachers: Hong Kong and Italy

**DOI:** 10.3389/fpsyg.2018.02626

**Published:** 2018-12-18

**Authors:** Paula Benevene, Yau Ho Paul Wong, Caterina Fiorilli, Simona De Stasio

**Affiliations:** ^1^Department of Human Studies, Libera Università Maria SS. Assunta, Rome, Italy; ^2^Department of Early Childhood Education, The Education University of Hong Kong, Tai Po, Hong Kong

**Keywords:** subjective well-being, mental health, kindergarten teachers, job satisfaction, self-esteem

## Abstract

**Background:** Teachers’ subjective well-being (SWB) has received much attention, in the light of the major increase in sick leave as well as job quitting among teachers across different cultures and countries. Studies on SWB of kindergarten teachers are still scarce, since most of the academic literature is focused on teachers of primary and secondary schools.

**Aims:** The purpose of this study was to analyze and compare kindergarten teachers’ SWB in Hong Kong and Italy.

**Methods:** 367 Hong Kong and 243 Italian kindergarten teachers completed a self-report questionnaire containing: the Job Satisfaction Survey (JSS), the Rosenberg Self-Esteem Scale (RSES), and the General Health Questionnaires-12 (GHQ-12). The questionnaire collected also socio-demographics data.

**Results:** Hierarchical regression analysis showed that country belonging plays the strongest predictive role on self-esteem and mental health. Moreover, the independent *t*-test showed higher levels of job satisfaction among Italian teachers, while Hong Kong teachers were more satisfied in pay and promotion, but less in supervision, operating condition, co-worker relationship, nature of work, and communication.

**Conclusion:** Results are interpreted in the light of the differences between the two contexts considered, in terms of cultural values and educational systems.

## Introduction

Teachers’ subjective well-being (SWB) has received much attention over the past decades, due to the major increase in sick leave as well as job quitting among teachers across different cultures and countries (Kyriacou and Sutcliffe, 1977; Hofstede, 1983; Kyriacou, 1987; Wong, 2010; Benevene and Fiorilli, 2015).

Teaching-related SWB refers to personal and work-related resources as well as the absence of physical and mental impairment, which generate a healthy functioning in the work environment (Bowling et al., 2010; Diener and Tov, 2012; Fiorilli et al., 2015; Benevene et al., 2018; Diener et al., 2018). Following Diener’s conceptualization, thus covering both cognitive and affective components as well as a specific domain satisfaction of SWB, the present study addresses teachers’ SWB through its three constructs of self-esteem (self or personality disposition, affective components), job satisfaction (focal domain satisfaction, cognitive components), and mental health complaints (cognitive and affective components).

Studies on teachers’ SWB have dealt almost exclusively with primary and secondary school teachers, whilst the literature on kindergarten teachers’ SWB has emerged as fragmented and mostly focusing on stress and burnout, deserving a more in-depth analysis (Bowling et al., 2010; Hall-Kenyon et al., 2014). In fact, the work of kindergarten teachers is physically demanding; teachers are required to nurse and mother the children, beyond their regular behavior management and instructional duties (McGrath and Huntington, 2007).

We thus developed an explorative, cross-country comparison between two groups of kindergarten teachers, respectively, from Italy and Hong Kong. We expected to find similarities as well as differences, and explained the latter in the light of the country contexts (Tov and Diener, 2007; Klassen et al., 2010).

The following hypotheses were developed:

Hypothesis 1: There is a positive correlation between teachers’ self-esteem and their job satisfaction, which in turn is negatively associated with teachers’ complaints about their mental health.
Hypothesis 2: Different national contexts play a predictive role on teachers’ SWB in both groups, even though their socio-demographic characteristics and years of teaching are taken into account.
Hypothesis 3: There are differences between kindergarten teacher groups in all SWB measures (job satisfaction, self esteem, mental health).

## Materials and Methods

### Participants

A quantitative cross-sectional method design was adopted. Two convenient samples of kindergarten teachers from Italy and Hong Kong were recruited from the metropolitan areas. Italian participants were 243: 95% female; 74% married; 43% had less than 10 years of teaching; 61% between 30 and 49-year-old and 28% above 50 years of age. Furthermore, 68% held a high school diploma, and 32% held a university degree.

Hong Kong participants were 371: 99% female; 57% married; 32% had less than 10 years of teaching experience; 73% was 30 to 49-year-old and 3% above 50; all participants had the early childhood education certificate approved by the government; 32% held a university degree.

### Instrument and Measures

A self-reported questionnaire was administered. The first part collected participants’ personal background: sex, age, marital status, and years of teaching. The second part contained:

#### The Job Satisfaction Survey (JSS)

The JSS measures respondents’ perceived satisfaction with their job situation (Spector, 1997). It consists of 36 items divided into nine subscales, namely: pay, promotion, supervision, fringe benefits, contingent rewards, operating procedures, co-workers, nature of work, and communication. The items were rated on a 5-point Likert response scale.

#### The Rosenberg Self-Esteem Survey (RSES)

The RSES is a one-dimensional instrument that measures global feeling of self-worth or self-acceptance using simple statements (Rosenberg, 1965). Participants were asked to rate their agreement with the proposed statements, based on a 4-point Likert response scale.

#### The General Health Questionnaire-12 (GHQ-12)

The GHQ-12 measures mental health complaints (Goldberg, 1972). It is based on the self-reported state of well-being among non-clinical populations in the previous 4 weeks. The reported symptoms and or behaviors were analyzed according to a 4-step answering scale.

The alpha coefficients for the Hong Kong version of the GHQ-12, RSES, and JSS were, respectively, 0.89, 0.84, and 0.90. Whereas for the Italian cohort, the alpha coefficients were, respectively, of 0.87 for the GHQ-12, 0.88 for the JSS, and 0.90 for the REES.

### Procedures

All participants gave their written informed consent before the administration of the questionnaire, in accordance with the 1964 Helsinki declaration.

Participants filled the questionnaire individually, which required about 20 min to be completed.

Our study did not need ethics approval, according to our national regulations and the Ethics Committee of the LUMSA University.

### Data Analysis

The Statistical Package for Social Sciences software version 18 was used to analyze the data. The Spearman rho correlation was performed to examine correlations among variables. Three multiple regression analyses were conducted to investigate the moderating effect of teachers’ nationality in the relation between their background variables and the SWB measures. Finally, the independent *t*-test was performed to verify possible statistical differences of SWB measures between the two country contexts.

## Results

Zero-order correlations, hierarchical regression analyses, and the independent *t*-test were performed as part of this study. However, the gender variable was excluded due to the imbalanced distribution, in favor of females, in both groups.

### Self-Esteem, Job Satisfaction, and Mental Health

Table 1 shows correlations between all of the studied variables in both groups. Among the Italian teachers, age shows weak associations with all SWB measures, with the exception of one JSS sub-scale (i.e., supervision), with which it has a positive and significant correlation (*r* = 0.27; *p* < 0.01). Years of teaching is significantly and negatively associated with GHQ (*r* = -0.27; *p* < 0.01), and positively associated with the following sub-scales of the JSS: supervision (*r* = 0.29; *p* < 0.01), co-workers (*r* = 0.20; *p* < 0.01), and communication (*r* = 0.23; *p* < 0.01). Teachers’ GHQ, as expected, has a significant and negative correlation with self-esteem (*r* = -0.27; *p* < 0.01) and with JSS (*r* = -0.37; *p* < 0.01). Supervision, co-workers and communication appear to be the most influential subscales on the entire JSS coefficient. In accordance with our hypothesis, teacher self-esteem is negatively associated with GHQ (*r* = -0.27; *p* < 0.01). Interestingly, and contrary to our hypotheses, no correlation emerged between teachers’ self-esteem and their Total Job Satisfaction.

**Table 1:** Pearson correlations between teachers’ background (age, years of teaching, marital status) and well-being variables.

	1	2	3	4	5	6	7	8	9	10	11	12	13	14	15
1. Age^#^	–	0.72^∗∗^	0.37^∗∗^	-0.12	0.15^∗^	0.06	-0.18^∗∗^	0.27^∗∗^	-0.07	0.04	-0.12	0.06	0.05	0.16^∗^	0.06
2. Years of teach	0.78^∗∗^	–	0.21^∗∗^	-0.27^∗∗^	0.11	0.01	-0.14^∗^	0.29^∗∗^	-0.08	0.04	-0.10	0.20^∗∗^	0.11	0.23^∗∗^	0.12
3. Marital	0.34^∗∗^	0.39^∗∗^	–	0.02	0.10	0.06	-0.10	0.04	-0.07	-0.09	-0.08	-0.03	0.07	0.02	-0.03
4. GHQ-12	-0.15^∗^	-0.19^∗∗^	-0.06	–	-0.27^∗∗^	-0.14^∗^	-0.13^∗^	-0.28^∗∗^	-0.15^∗^	-0.23^∗∗^	-0.18^∗∗^	-0.33^∗∗^	-0.18^∗∗^	-0.36^∗∗^	-0.37^∗∗^
5. Self esteem	0.14^∗^	0.13^∗^	0.14^∗∗^	-0.45^∗∗^	–	-0.11	-0.09	0.22^∗∗^	-0.09	0.04	-0.03	0.06	0.18^∗∗^	0.18^∗∗^	0.07
6. Pay	0.04	0.11	0.02	-0.13^∗^	0.08	–	0.43^∗∗^	0.11	0.55^∗∗^	0.50^∗∗^	0.27^∗∗^	0.29^∗∗^	0.02	0.18^∗∗^	0.63^∗∗^
7. Promotion	0.19^∗∗^	0.24^∗∗^	0.10	-0.07	0.19^∗∗^	0.36^∗∗^	–	0.01	0.57^∗∗^	0.34^∗∗^	0.31^∗∗^	0.14^∗^	-0.11	-0.01	0.49^∗∗^
8. Supervision	0.13	0.19^∗∗^	0.12^∗^	-0.17^∗^	0.27^∗∗^	0.39^∗∗^	0.40^∗∗^	–	0.15^∗^	0.35^∗∗^	0.26^∗∗^	0.42^∗∗^	0.26^∗∗^	0.47^∗∗^	0.58^∗∗^
9. Benefits	0.07	0.18^∗^	0.06	-0.17^∗^	0.01	0.72^∗∗^	0.38^∗∗^	0.44^∗∗^	–	0.55^∗∗^	0.36^∗∗^	0.27^∗∗^	0.02	0.18^∗∗^	0.67^∗∗^
10. Rewards	0.12	0.20^∗∗^	0.06	-0.16^∗^	0.24^∗∗^	0.54^∗∗^	0.41^∗∗^	0.68^∗∗^	0.47^∗∗^	–	0.36^∗∗^	0.39^∗∗^	0.12	0.45^∗∗^	0.76^∗∗^
11. Conditions	0.13	0.11	0.03	-0.27^∗∗^	0.06	0.38^∗∗^	0.13^∗^	0.41^∗∗^	0.39^∗∗^	0.47^∗∗^	–	0.28^∗∗^	0.07	0.24^∗∗^	0.58^∗∗^
12. Coworkers	-0.08	-0.06	0.02	-0.17^∗^	0.08	0.09	0.13	0.45^∗∗^	0.20^∗∗^	0.33^∗∗^	0.18^∗^	–	0.21^∗∗^	0.55^∗∗^	0.68^∗∗^
13. Nature work	0.13	0.07	0.05	-0.19^∗∗^	0.24^∗∗^	0.13	0.13^∗^	0.33^∗∗^	0.13	0.23^∗∗^	0.28^∗∗^	0.27^∗∗^	–	0.24^∗∗^	0.32^∗∗^
14. Communication	0.04	0.15^∗^	0.14^∗∗^	-0.11	0.19^∗∗^	0.35^∗∗^	0.33^∗∗^	0.70^∗∗^	0.36^∗∗^	0.65^∗∗^	0.36^∗∗^	0.53^∗∗^	0.32^∗∗^	–	0.63^∗∗^
15. TJS	0.13^∗^	0.21^∗∗^	0.09	-0.23^∗∗^	0.23^∗∗^	0.71^∗∗^	0.57^∗∗^	0.81^∗∗^	0.72^∗∗^	0.81^∗∗^	0.60^∗∗^	0.49^∗∗^	0.44^∗∗^	0.74^∗∗^	–


*Correlation figures above and below the diagonal refer to Italy and Hong Kong teachers, respectively. ^#^Age group: 1: <30 years old; 2: 30–39 years old; 3: 40–49 years old; 4: >50 years old. TJS, total job satisfaction, ^∗^*p* < 0.05; ^∗∗^*p* < 0.01.*

In the Hong Kong context, years of teaching is positively correlated with JSS (*r* = 0.21; *p* < 0.01) through the significant contribution of two of its subscales: promotion and rewards. As expected, GHQ also shows significant and negative correlations with teachers’ self-esteem (*r* = -0.45; *p* < 0.01) and JSS (*r* = -0.23; *p* < 0.01), with a relevant contribution of the “conditions” subscale (Table 1). Furthermore, teachers’ self-esteem is positively associated with JSS (*r* = 0.23; *p* < 0.01), with a major contribution by the “supervision,” “rewards,” and “nature of work” subscales.

### Kindergarten Teachers’ Socio-Demographics, Country and SWB

Table 2 shows the findings of the three multiple regressions models, conducted to examine whether the national context influenced the relation between the socio-demographic variables and the three dimensions of SWB. The predictors were entered in the first step and interaction terms were entered in the second step. The first regression analysis (*R*^2^= 0.03, *p* < 0.01) revealed that the variables entered explained 3% of the variance of job satisfaction. Our findings highlight that national context has a weak contribution on job satisfaction (β = -0.13, *p* < 0.01).

**Table 2:** Multiple regression analysis.

	Teachers’ well-being					
	Job satisfaction		GHQ-12		Self-esteem	
Variables	β	*R*^2^	β	*R*^2^	β	*R*^2^
		0.03^∗∗^		0.28^∗∗^		0.30^∗∗^
Age	0.04		0.09		0.06	
Years of teaching	0.07		-0.18^∗∗^		0.02	
Marital status	-0.08		0.00		0.03	
Culture^∗^	-0.13^∗∗^		-0.47^∗∗^		-0.52^∗∗^	
		0.03		0.29		0.31
Age × culture	0.04		-0.12		-0.04	
Years of teaching × culture	0.04		0.02		0.13	


*^∗∗^*p* < 0.01. ^∗^Culture was considered a dummy variable. Italian teachers were the reference group.*

From the second regression analysis, we find a significant model predicting teachers’ mental health complaints (*R*^2^= 0.28, *p* < 0.01). However, the strongest principal effect is due to teachers’ nationality (β = -0.47; *p* < 0.01), with more mental health complaints among Italians. There is instead a weak effect of teachers’ years of experience on mental health complaints (β = -0.18; *p* < 0.01). Finally, the third regression model (*R*^2^ = 0.30, *p* < 0.01) shows that teachers’ national context is a significant predictor of Self-Esteem (β = -0.52; *p* < 0.01): it increases the level of self-esteem among Italians. No significant interactions were found between national context and socio-demographic characteristics on teachers’ SWB.

### Differences Between Italy and Hong Kong About SWB

Table 3 shows means, standard deviations, and mean differences between the two groups of kindergarten teachers on their job satisfaction’s subscales. The independent *t*-test values show significant differences on different subscales.

**Table 3:** Mean differences between Italian teachers (*n* = 242) and Hong Kong teachers (*n* = 371).

	Italy	Hong Kong		
Variable	Mean (SD)	Mean (SD)	Difference in mean	*t*-Value
Pay	8.72 (4.25)	12.86 (4.86)	-4.15	-11.12^∗∗^
Promotion	9.98 (4.1)	11.8 (3.83)	-1.82	-5.55^∗∗^
Supervision	18.81 (4.39)	14.35 (4.23)	4.46	12.55^∗∗^
Benefits	11.14 (3.79)	11.69 (4.19)	-0.55	-1.67
Rewards	13.28 (4.25)	13.08 (3.77)	0.21	0.61
Conditions	13.46 (3.82)	11.09 (3.34)	2.38	7.90^∗∗^
Co-workers	17.3 (4.37)	16.12 (3.13)	1.18	3.64^∗∗^
Nature work	20.65 (3.44)	17.95 (3.21)	2.70	9.90^∗∗^
Communication	16.26 (4.54)	14.73 (3.71)	1.53	4.37^∗∗^
TJS	129.61 (22.13)	123.89 (22.66)	5.72	3.06^∗∗^


*TJS, Total job satisfaction, ^∗∗^*p* < 0.01.*

More specifically, Italian kindergarten teachers show a higher level of job satisfaction (*t* = 3.06, *p* < 0.01) and self-esteem (*t* = 15.67, *p* < 0.01) than their counterparts in Hong Kong, even though they are less satisfied with their pay (*t* = -11.12, *p* < 0.01) and promotion opportunities (*t* = -5.55, *p* < 0.01).

## Discussion

Our findings highlight that country context can predict mental health and self-esteem. More specifically, correlational findings in both groups showed positive correlations between age and job satisfaction linked to promotion (Ingusci, 2018). This is not surprising as opportunities of promotion increase with time in both countries. Furthermore, amongst the Hong Kong cohort, years of teaching are positively related with promotion and reward, since this school system offers teachers real opportunities in this regard (Li, 2009). On the other hand, amongst the Italian teachers there are significant correlations between years of teaching and supervision, quality of communication with colleagues and relationships with co-workers. Actually, the Italian school system foresees a big deal of planning class activities among colleagues, requiring a strong co-ordination among teachers. This sort of teamwork attitude develops with time, if properly monitored and nurtured (Gardner and Pierce, 2016; De Stasio et al., 2017) and furthermore may explain why Italian teachers show a positive correlation between self esteem and three sub-scales of the JSS: supervision, nature of work, and communication.

Moreover, there are significant and positive correlations among Hong Kong teachers’ self-esteem and job satisfaction, while in the Italian context these variable are unrelated, probably because salary and promotion of Italian teachers depends on seniority, rather than on merit (Leigh and De Vogli, 2016).

### Socio-Demographics and SWB Dimensions

In both groups, self-esteem is negatively associated with mental health complaints, while job satisfaction is significantly correlated with mental health. Thus, in both contexts job satisfaction and self-esteem seem to protect teachers from risks of mental ill-being.

Conversely, teachers’ age and marital status are not correlated with the three components of SWB in either group, and years of teaching is related with just some of the studied variables in both contexts.

### Socio-Demographics, Country Context, and SWB

Findings of the regression analyses highlight that teachers’ national context contributes in both groups to kindergarten teachers’ self-esteem and mental health. Furthermore, country context does not appear to significantly moderate the relationship between teachers’ background variables and the SWB dimensions. Specifically, Italian teachers show higher levels of self-esteem whilst having more mental health complaints. These results are in line with a previous study, which found that Chinese teachers regulate their emotions by “genuinely expressing their feelings, along with surface acting and deep acting, therefore developing more resilience” (Yin, 2015, p. 20). The stronger resilience among Hong Kong teachers may lead them to express less mental health complaints, despite their lower self-esteem in comparison to their Italian colleagues. In fact, the values of any given society are related to the behavior of its members (Hofstede, 1983).

### Differences Between Italy and Hong Kong on Job Satisfaction’s Subscales

Italian teachers show higher mean scores on total job satisfaction and in several specific facets (supervision, work conditions, relationships with co-workers, the nature of work, and communication). These findings, again, may be read in the light of the strong co-operation required by the Italian kindergarten teachers within their working environment. In fact, experiencing positive relationships at work and a supportive environment has proven to be related to both job satisfaction and self-esteem (Gardner and Pierce, 2016). At the same time, however, Italian teachers show lower mean scores in terms of pay and promotion opportunities. In fact Italian teachers have a lower annual base salary compared to other European countries and are more limited in the career advancement paths open to them (Organisation for Economic Co-operation and Development [OECD], 2013). This suggests that pay and promotion may play a role in determining the global level of job satisfaction, even though these may be counterbalanced by the effects of other job-satisfaction dimensions, as the Italian teachers’ scores suggest (Borrelli et al., 2014; McInerney et al., 2015; Buonomo et al., 2017; Fiorilli et al., 2017).

### Limitations and Future Line of Research

Participants do not represent a statistically representative sample. Future research might replicate the present study in other Western and Asian countries, with a representative sample, to verify whether our results are confirmed in different national contexts, considering also other variables, such as job insecurity. Furthermore, since teachers of non-profit kindergartens tend to show higher levels of well-being, it would worth to observe different types of school (Wong, 2010).

## Author Contributions

All authors contributed at the same extent to the research project, data analyst as well as the writing of the manuscript. YW collected the data in Hong Kong. PB, CF, and SDS collected the data in Italy.

## Conflict of Interest Statement

The authors declare that the research was conducted in the absence of any commercial or financial relationships that could be construed as a potential conflict of interest.
